# An Improved Deep Reinforcement Learning Method for Dispatch Optimization Strategy of Modern Power Systems

**DOI:** 10.3390/e25030546

**Published:** 2023-03-22

**Authors:** Suwei Zhai, Wenyun Li, Zhenyu Qiu, Xinyi Zhang, Shixi Hou

**Affiliations:** 1 Electric Power Research Institute of China Southern Power Grid Yunnan Power Grid Co., Ltd., Kunming 650217, China; 2 Yunnan Power Dispatching Control Center of China Southern Power Grid, Kunming 650011, China; 3 College of IOT Engineering, Hohai University, Nanjing 210098, China

**Keywords:** wind farm, energy storage system, reinforcement learning, deep neural networks

## Abstract

As a promising information theory, reinforcement learning has gained much attention. This paper researches a wind-storage cooperative decision-making strategy based on dueling double deep Q-network (D3QN). Firstly, a new wind-storage cooperative model is proposed. Besides wind farms, energy storage systems, and external power grids, demand response loads are also considered, including residential price response loads and thermostatically controlled loads (TCLs). Then, a novel wind-storage cooperative decision-making mechanism is proposed, which combines the direct control of TCLs with the indirect control of residential price response loads. In addition, a kind of deep reinforcement learning algorithm called D3QN is utilized to solve the wind-storage cooperative decision-making problem. Finally, the numerical results verify the effectiveness of D3QN for optimizing the decision-making strategy of a wind-storage cooperation system.

## 1. Introduction

Since the beginning of the 21st century, higher requirements for energy conservation, emission reduction, and sustainable development have been put forward as a result of the increasing pressure from the use of global resources. Thus, clean energy has gained much attention, which further accelerates the global energy transformation [1,2,3]. At present, the commonly used clean energy sources include wind energy, solar energy, and tidal energy. Among these clean energy sources, wind energy outperforms with its rich resources, low cost, and relatively mature technology [4,5].

However, because of the great correlation between wind energy and environmental information, its power generation is characterized by randomness, uncontrollability, and volatility, which seriously affects the power balance and threatens the stable and safe operation [6]. Equipping the wind farm with an energy storage system can alleviate the above problems to a certain extent [7,8,9,10]. Therefore, how to realize a high-efficient wind-storage cooperative decision-making is a key issue for promoting the full absorption of wind energy [11,12].

Reinforcement learning, also known as a promising information theory, is a machine learning method based on environmental feedback information [13,14]. Its decision theory is very suitable for issues containing complex environments and multiple variables. At present, some studies have proven the feasibility and effectiveness of the energy allocation strategy using reinforcement learning in the field of power system, such as load frequency control on the generation side and market competition strategy [15,16,17,18].

Despite several works that have proposed reinforcement learning methods for wind-storage cooperative decision-making, some issues still exist, as follows:

(1) The flexible loads embedded in the wind-storage cooperative framework have not been developed sufficiently in the existing literature. In [11,19,20,21], the authors did not focus on the favorable effect of the flexible loads in the proposed wind-storage model. As an example, flexible loads were considered in [22], where the benefits from the suitable management of demand-side flexible loads were validated. However, the detailed formula for when the load in the price response load model should be shifted was not given.

(2) The exploration of reinforcement learning methods for wind-storage cooperative decision-making needs to be enhanced. In [19,20,23,24], a deep Q-learning strategy was considered in wind-storage systems. However, the main mechanism of the deep Q-learning strategy is to select the actions that can obtain the maximum benefits according to the Q values, which are constructed by the state and action. It has been reported that using the same networks to generate the Q values and its maximum estimated value will result in the maximizing deviation issue, which tends to deteriorate the network accuracy.

Motivated by the above analysis, a novel wind-storage cooperative decision-making model including demand-side flexible loads is developed in this paper, which comprehensively considers the direct or indirect control of various power components, improves the reasonable allocation ability of the energy controller, and enhances the economy and stability of the power grid. Moreover, in order to tackle the defects of the traditional deep Q-learning method, the dueling double deep Q-network (D3QN), which is constructed by two networks (the evaluation network and target network), is developed for the wind-storage cooperative decision-making control mechanism in this study.

The remainder of this study is organized as follows: wind-storage cooperative model and D3QN are presented in Section 2. In Section 3, the wind-storage cooperative decision-making algorithm using D3QN is presented. The algorithm evaluation details and the numerical results are presented in Section 4 and Section 5. Section 6 presents the conclusions.

## 2. Wind-Storage Cooperative Model and D3QN

### 2.1. Wind-Storage Cooperative Decision-Making Model

This study mainly focusses on a wind-storage cooperative model, including wind turbines and energy storage systems, which also is connected to the external power grid.

**Figure 1:**
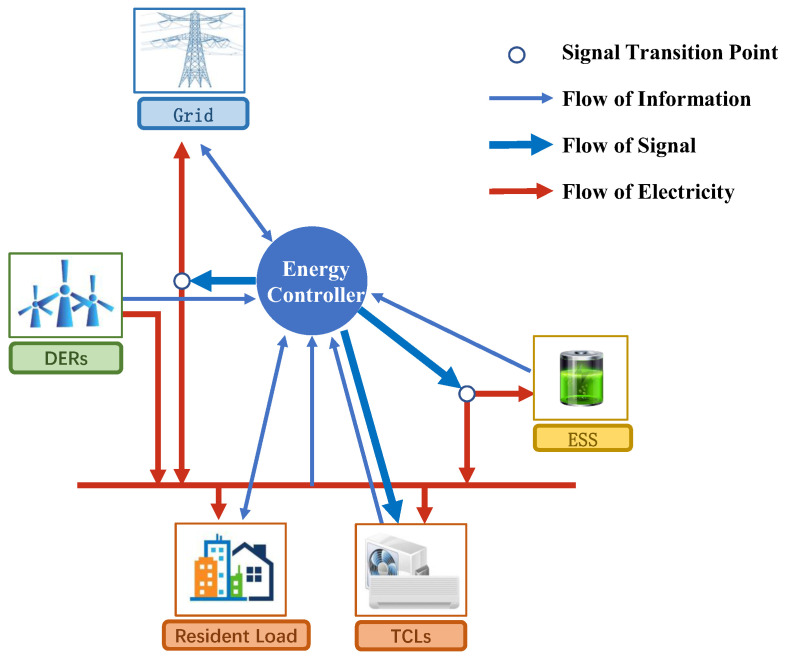
Wind-storage cooperative model.

The architecture of the wind-storage cooperative model is shown in Figure 1. Three layers exist: the electricity layer, information layer, and signal layer. The electricity layer includes a distributed energy resources (DER) based on wind power, an energy storage system (ESS) for the storage and release of wind power energy, a group of thermostatically controlled loads (TCLs), and a group of price responsive loads. The information layer is composed of a two-way communication system between the external power grid, each power module, and the energy controller (EC). Information such as electricity price, as well as the battery charge and discharge status are transmitted in the information layer. The signal layer transmits the control signals sent by the energy controller to each controllable module. The whole system model has three direct control points, namely, the switch control of TCLs, the charging and discharging control of ESS, and the trading control of energy on the external power grid.

At the same time, the whole wind-storage cooperative model can also be regarded as a multi-agent system. Each module in the system is regarded as an autonomous agent, which can interact with the environment and other agents. Moreover, the simple or complex behavior of each agent is controlled by its internal model. The models used in each module of the whole wind-storage cooperation model will be introduced in detail below.

#### 2.1.1. External Power Grid

Because of the intermittent and uncontrollable characteristics of DER, the use of DER alone may not be able to balance the relationship between supply and demand in the power grid. Therefore, the external power grid is considered as the regulatory reserve in this system model. The external power grid can provide electric energy immediately when the wind-storage energy is insufficient, and the external power grid can also accept the excess electricity when the wind energy is in surplus. The transaction price is defined by the real-time price in the power market. The market prices are expressed as $\left(P_{t}^{u},P_{t}^{d}\right)$, where $P_{t}^{u}$ and $P_{t}^{d}$ represent the increased and decreased price, respectively.

#### 2.1.2. Distributed Energy Module

Wind turbines are considered as the distributed energy equipment in this study. Specifically, actual wind data from a wind farm in Finland [25] are directly used to construct the model of DER. DER shares the currently generated electric energy information $G_{t}$ with the energy controller.

#### 2.1.3. Energy Storage System Module

In order to reasonably optimize the allocation of energy and reduce the cost of energy consumption, this study uses the community energy storage system, rather than a separate household storage battery. As a centralized independent energy storage power station invested by a third party, the community energy storage system can integrate and optimize the allocation of the dispersed energy storage resources from the power grid side, power supply side, and user side.

For each time step t, the dynamic model of ESS is defined as follows [26]:

(1) $$B_{t}=B_{t-1}+\eta_{c}C_{t}-\frac{D_{t}}{\eta_{d}}$$

where $B_{t}\;\;\in [0,B_{\max}]$ is the electric energy stored by ESS at time t, and $B_{\max}$ is the maximum storage capacity of ESS. $\eta_{c}$ and $\eta_{d}$ are the charging and discharging efficiency coefficients of energy storage equipment, respectively, and $(\eta_{c},\eta_{d}\;)\in {\left(0,1\right]}^{2}$. The variables $C_{t}\in [0,C_{\max}]$ and $D_{t}\in [0,D_{\max}]$ represent charge and discharge power, respectively, which are limited by the maximum charge and discharge rate $C_{\max}$ and $D_{\max}$ of ESS, respectively.

The state-of-charge variable of ESS is defined as BEC:

(2) $$BEC_{t}=\frac{B_{t}}{B_{\max}}\times 100\%$$

When the energy controller releases the charging signal, ESS obtains the current electricity stored in the battery and verifies the feasibility of the charging operation by referring to the maximum storage capacity $B_{\max}$ and the maximum charging rate $C_{\max}$. Then, ESS stores the corresponding electricity according to the actual situation and the remaining excessive electricity will be sold to the external power grid. When ESS receives the discharge signal, it verifies the relevant conditions again to judge the operational feasibility and provides the electricity accordingly. If ESS cannot fully provide the requested electricity, the insufficient part will be automatically provided by the external power grid, and the agent will need to pay the relevant costs.

#### 2.1.4. Thermostatically Controllable Load

Thermostatically controllable loads (TCLs) are characterized by their large size, flexible control, and energy conservation. In this study, it is assumed that the vast majority of households are equipped with TCLs, such as air conditioners, water heaters, and refrigerators. These TCLs can be directly controlled in each time unit t and the control signal comes from the TCL aggregator. As EC directly controls TCL equipment, this study defines that TCL will only be charged for power generation costs $C_{gen}$ in order to compensate TCL users. To maintain the comfort of users, each TCL is equipped with a backup controller, which can keep the temperature within an acceptable range. The backup controller receives the on/off operation $u_{t}^{i}$ from the TCL aggregator and modifies its action by verifying the temperature constraints. The specific definitions are as follows:

(3) $$u_{b,t}^{i}=\{\begin{array}{ccc} 0 & if & T_{t}^{i}>T_{\max}^{i}\\ u_{t}^{i} & if & T_{\min}^{i}<T_{t}^{i}<T_{\max}^{i}\\ 1 & if & T_{t}^{i}<T_{\min}^{i} \end{array}$$

where $u_{b,t}^{i}$ is the on/off action of the ith TCL backup controller at t, $T_{t}^{i}$ is the operating temperature of the ith TCL at t, and $T_{\max}^{i}$ and $T_{\min}^{i}$ are the upper and lower temperature boundaries set by the client, respectively. The differential equation of the temperature change in the building is designed as follows [27]:

(4) $${\dot{T}}_{t}^{i}=\frac{1}{C_{a}^{i}}(T_{t}^{0}-T_{t}^{i})+\frac{1}{C_{m}^{i}}\;(T_{m,t}^{i}-T_{t}^{i})+L_{TCL}^{i}u_{b,t}^{i}+q^{i}$$

(5) $${\dot{T}}_{m,t}^{i}=\frac{1}{C_{m}^{i}}(T_{t}^{i}-T_{m,t}^{i})$$

where $T_{t}^{i}$, $T_{m,t}^{i}$, and $T_{t}^{0}$ are the indoor air temperature, indoor solid temperature, and outdoor air temperature at t, respectively, $C_{a}^{i}$ and $C_{m}^{i}$ are expressed as the equivalent heat capacity of indoor air and solid, respectively, $q^{i}$ is the thermal power provided by indoor temperature control equipment, and $L_{TCL}^{i}$ is the rated power of TCL.

Finally, the state of charge (SoC) is used to represent the relative position of the current temperature $T_{t}^{i}$ within the expected temperature range. The SoC of each TCL at t is defined as follows:

(6) $$SoC_{t}^{i}=\frac{\left(T_{t}^{i}-T_{\min}^{i}\right)}{\left(T_{\max}^{i}-T_{\min}^{i}\;\right)}$$

#### 2.1.5. Resident Price Response Load

Some power demands exist from household that the energy controller cannot directly control in the residential load [28]. This study assumes that the daily electricity consumption of residents is composed of the daily basic electricity consumption and the flexible load affected by the electricity price. The flexible load can operate in advance or later within the acceptable time range and can be transferred according to the power generation situation of DER, such that the resource utilization rate can be improved and the household electricity expenditure can also be reduced. In this module, each household i has a sensitivity factor $\beta_{i}\in \left(0,1\right)$ and a patience parameter $\lambda_{i}$, in which the sensitivity factor β represents the percentage of load that can be operated in advance or later when the price decreases or increases, and the patience parameter λ represents the hours to repay the transferred load. For example, when the electricity price is high, this part of the load can be cut now and operated after $\lambda_{i}$.

At t, the load $L_{t}^{i}$ of household i is modeled by the following formula:

(7) $$L_{t}^{i}=L_{b,t}-SL_{t}^{i}+PB_{t}^{i}$$

(8) $$SL_{t}^{i}=L_{b,t}*\beta_{i}*\delta_{t}$$

where $L_{b,t}$ represents the daily basic load of residents, $L_{b,t}>0$, and $L_{b,t}$ follows the daily consumption pattern, which can be inferred from the average daily consumption curve of residential areas. $SL_{t}^{i}$ is the shift load (SL) defined by (8), where $\delta_{t}$ represents the electricity price level at t. Therefore, $SL_{t}^{i}$ is positive when the price is high, i.e., $\delta_{t}>0$, then $SL_{t}^{i}>0$, and when the price is low, i.e., $\delta_{t}<0$, then $SL_{t}^{i}<0$. The positive transfer load will be repaid after a certain period of time λ. The negative transfer load is the electricity provided in advance, so it will exist in the future. The loads to be compensated can be formulated as follows:

(9) $$PB_{t}^{i}=\;\sum_{j=0}^{t-1}\omega_{i,j}*SL_{j}^{i}$$

where $\omega_{i,j}\in \{0,1\}$ represents the compensation degree for the transferred load at j. Generally, the closer t minus j is to $\lambda_{i}$, the higher $\omega_{i,j}$ is. In addition, the compensation action also should be related to the electricity price, i.e., $\omega_{i,j}$ becomes smaller when $\delta_{t}>0$. Therefore, $\omega_{i,j}$ can be designed as follows:

(10) $$\omega_{i,j}=clip\left(\frac{-\delta_{t}*sign(SL_{j}^{i})}{2}+\frac{t-j}{\lambda_{i}},0,1\right)$$

(11) $$clip(X,a,b)=\{\begin{array}{ccc} a & if & X<a\\ X & if & a\leq X\leq b\\ b & if & X>b \end{array}$$

Given (10), when $\delta_{t}>0$, one can obtain $SL_{t}^{i}>0$ and $sign(SL_{j}^{i})>0$, then $\frac{-\delta_{t}*sign(SL_{j}^{i})}{2}<0$, which means that $\omega_{i,j}$ becomes smaller and the positive transfer load almost cannot be compensated in the case of a high price [29,30].

#### 2.1.6. Energy Controller

**Figure 2:**
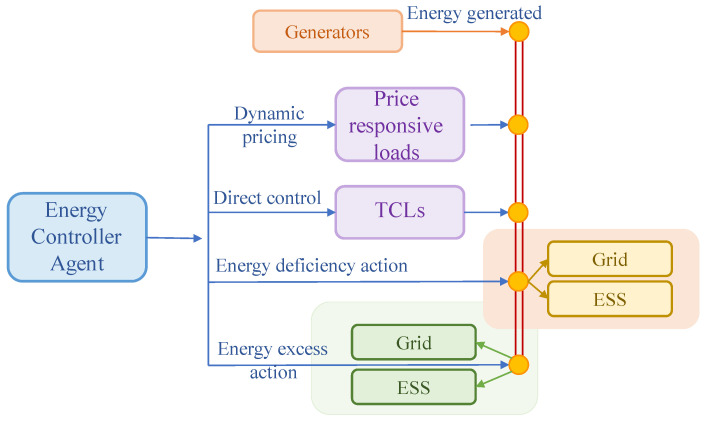
The control mechanism of Energy controller.

In this study, EC can extract the information provided by different modules and the observable environment to determine the best supply and demand balance strategy. EC mainly manages the power grid through four control mechanisms, as shown in Figure 2, including TCL direct control, price level control, energy deficiency action, and energy excess action.

(1) TCL direct control

**Figure 3:**
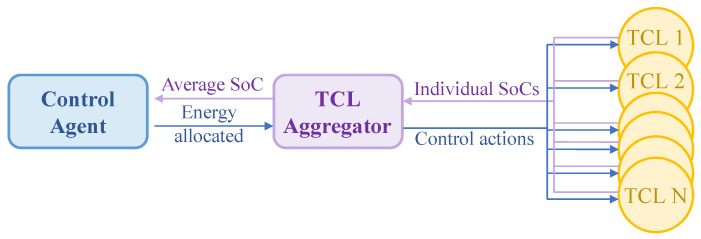
The intermediary role of the TCL aggregator.

At each time step t, EC will allocate a certain amount of electric energy for TCLs. Then, they will be distributed to each TCL through a TCL aggregator. The TCL aggregator judges the priority of energy distribution according to the power delivered by EC and the SoC of each TCL, and then determines the on/off action of each TCL: TCL with a lower SoC has a higher priority in energy allocation than TCL with a higher SoC. The TCL aggregator also operates as an information aggregator transmitting the real-time average SoC information of the TCL cluster to EC [31]. The specific transmission process is shown in Figure 3.

(2) Price level control

In order to effectively utilize the elastic benefits of the residential price response load, EC must determine the electricity price level $\delta_{t}$ at each time step t. In order to ensure the competitiveness of the system model proposed in this paper, a pricing mechanism is designed: The price can fluctuate around the median value, but the average price of the daily electricity price $P_{avg}$ cannot exceed 2.9% of the market electricity price provided by power retailers [32]. From a practical point of view, the electricity price at the DR side is discrete, and its fluctuation is affected by the electricity price level $\delta_{t}$. So, the real-time electricity price is selected from five values:

(12) $$P_{t}\in (P_{market}+\delta_{t}*cst)$$

where $\delta_{t}\in \{-2,-1,0,1,2\}$, cst is the constant to determine the specific increment or reduction in electricity price.

In addition, the model also pays attention to the electricity price level $\delta_{t}$ at each moment. When the sum of the previous electricity price levels is higher than the set threshold, the market electricity price is adjusted to $P_{market}$ instead of the price given by the agent. The effective electricity price level $\delta_{t,eff}$ is defined as follows:

(13) $$\delta_{t,eff}=\{\begin{array}{c} \begin{array}{ccc} \delta_{t} & if & \sum_{j=0}^{t}\delta_{t}\leq threshold \end{array}\\ \begin{array}{ccc} 0 & if & \sum_{j=0}^{t}\delta_{t}>threshold \end{array} \end{array}$$

(3) Energy deficiency action

When the power generated from DER cannot meet the power demand, EC can dispatch the energy stored in ESS or purchase energy from an external power grid. EC will determine the energy priority between ESS and an external power grid. In addition, if the high priority energy is ESS but the electricity stored in ESS cannot meet the power demand, the remaining power will be automatically supplied by an external power grid.

(4) Energy excess action

When the electricity generated by local DER exceeds the electricity demand, the excess electricity must be stored in ESS or be sold to an external power grid. In this case, EC also will determine the priority between ESS and the external power grid. If ESS is the preferred option and it has reached the max capacity, the remaining electricity will be automatically transmitted to an external power grid.

### 2.2. D3QN

In this section, the basic principle of DQN (deep Q-network) and SARSA (state−action−reward−state−action) is presented first.

The train mechanism of DQN can be formulated as follows:

(14) $$Q_{k+1}(s,a)=Q_{k}(s,a)+\alpha E_{k}$$

(15) $$E_{k}=R+\gamma argmax_{a^{\prime }}Q(s^{\prime },a^{\prime })-Q(s,a)$$

Using (14), one can find that the update iteration needs to achieve the approximation of the action-value function value (i.e., $Q_{k+1}(s,a)=Q_{k}(s,a)$), which means $R+\gamma argmax_{a^{\prime }}Q(s^{\prime },a^{\prime })-Q(s,a)\to 0$. Thus, the DQN network parameters can be updated by minimizing the mean square error loss function in the DQN algorithm.

The difference in the SARSA algorithm lies in how the Q value is updated. Specifically, when the agent with the SARSA algorithm is in the state s, it selects the action a according to the ε−greedy, and then observes the next state $s^{\prime }$ from the environment, and selects the action $a^{\prime }$ again. The sequence $\left\{s,a,r,s^{\prime },a^{\prime }\right\}$ is stored in the empirical replay set, and the calculation of the target Q value also depends on it. The core idea of the SARSA algorithm can be simplified as follows:

(16) $$Q(s,a)\leftarrow Q(s,a)+\alpha [R+\gamma \arg\max_{a^{\prime }}Q(s^{\prime },a^{\prime })-Q(s,a)]$$

**Figure 4:**
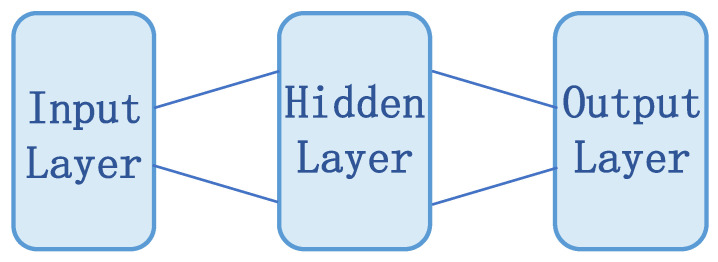
The network structure of DQN.

**Figure 5:**
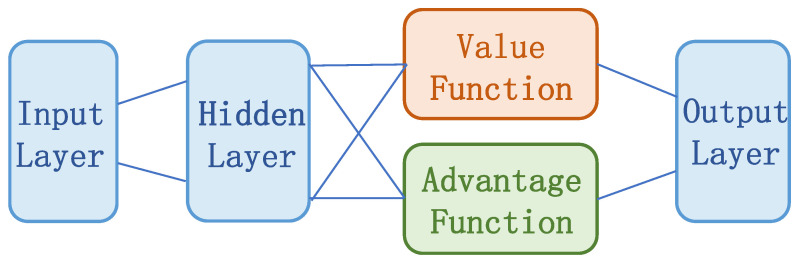
The network structure of D3QN.

In the existing study, DQN and SARSA have been developed for the wind-storage cooperative decision-making algorithm. However, both DQN and SARSA use Q(s,a) and $maxQ(s^{\prime },a^{\prime })$ produced by the same network to update the Q network parameter ω, which leads to the variation in the timing difference goal and a reduction in the convergence performance. Therefore, in view of the above possible problems, this paper uses the D3QN algorithm to optimize the model decision. The specific improvements are collected as follows:

(1) Referring to the double DQN (DDQN) algorithm, two neural networks with the same structure are constructed as the estimation network Q(s,a,ω) and the target network $Q^{\prime }(s,a,\omega^{\prime })$, respectively. The estimation network is used to select the action corresponding to the maximum Q value, and its network parameters are constantly updated. The target network is used to calculate the target value y, and its network parameters are fixed, but they are updated by using the current estimated network parameters value at regular intervals. The parameters in the target network are fixed for a period of time, which makes the convergence target of the estimated network relatively fixed, which is beneficial to the convergence of the algorithm model, and also avoids the agent selecting the overestimated suboptimal action. The overestimation problem of the DQN algorithm can also be effectively solved.

(2) In this paper, the structure of the deep neural network is adjusted. Referring to dueling DQN based on competitive architecture, the main output is divided into two parts: one part is the state-value function V(S,ω,α), which represents the current state; the other part is the advantage function A(S,A,ω,β), which judges the additional value level of each action for the current state. The neural network structure of DQN is shown in Figure 4, and the neural network structure of D3QN is shown in Figure 5.

Finally, the output of the Q network is obtained by the linear combination of the output of the state-value function network and the advantage function network:

(17) Q(S,A,ω,α,β)=V(S,ω,α)+A(S,A,ω,β)

However, (17) cannot identify the respective functions of V(S,ω,α) and A(S,A,ω,β) in the final output. In order to reflect this identifiability, the advantage function is generally set as the single action advantage function minus the average value of all of the action advantage functions in a certain state, so it can be modified as follows:

(18) $$\begin{array}{l} Q(S,A,\omega ,\alpha ,\beta )=V(S,\omega ,\alpha )+\\ \;\;\;\;\;\;\;\;\;\;\;\;\;\;\;\;\;\;\;\;\;\;\;\;\;\;\;\;\;\;A(S,A,\omega ,\beta )-\frac{1}{A}\sum_{a^{\prime }\in A}A(S,a^{\prime },\omega ,\beta ) \end{array}$$

**Figure 6:**
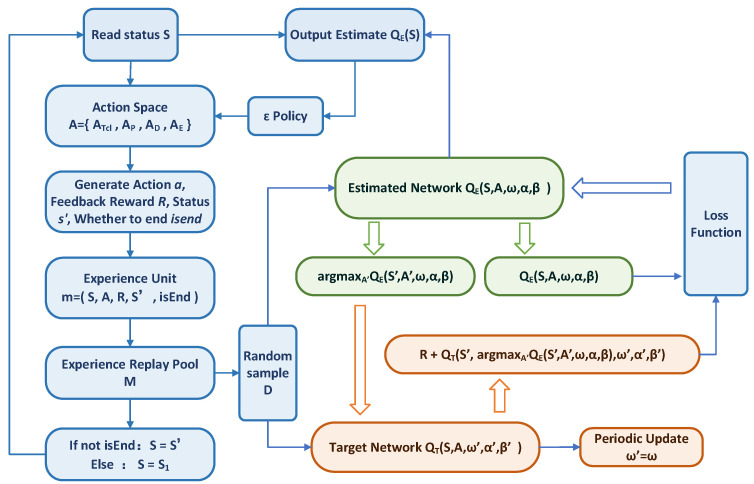
The flow chart of D3QN.

The flow chart of D3QN is shown in Figure 6:

In Figure 6, the D3QN algorithm stores the experience gained from the interaction in the experience pool one by one. After a certain amount is accumulated, the model randomly extracts a certain batch of data from the experience pool in each step to train the neural network. These randomly extracted experiences break the correlation between data, improve the generalization performance, and benefit from the stability of network training. Meanwhile, in Figure 6, the D3QN algorithm constructs two neural networks with the same structure, namely, the estimated network $Q_{E}(S,A,\omega ,\alpha ,\beta )$ and the target network $Q_{T}(S,A,\omega^{\prime },\alpha^{\prime },\beta^{\prime })$. The estimated network is used to select the action and parameter ω is updated constantly. The target network is used to calculate the temporal difference of the target value. Parameter $\omega^{\prime }$ is fixed and replaced with the latest estimated network parameter ω at regular intervals. $\omega^{\prime }$ remains unchanged for a period of time, resulting in a relatively fixed convergence goal of the estimated network $Q_{E}$, which is beneficial for convergence. The actions of the maximum function generated by the estimated network and the target network are not necessarily the same. Using $Q_{E}$ to generate actions and $Q_{T}$ to calculate the target value can prevent the model from selecting the overestimated sub-optimal actions and can effectively solve the overestimation problem of the DQN algorithm.

## 3. Wind-Storage Cooperative Decision-Making Based on D3QN

**Figure 7:**
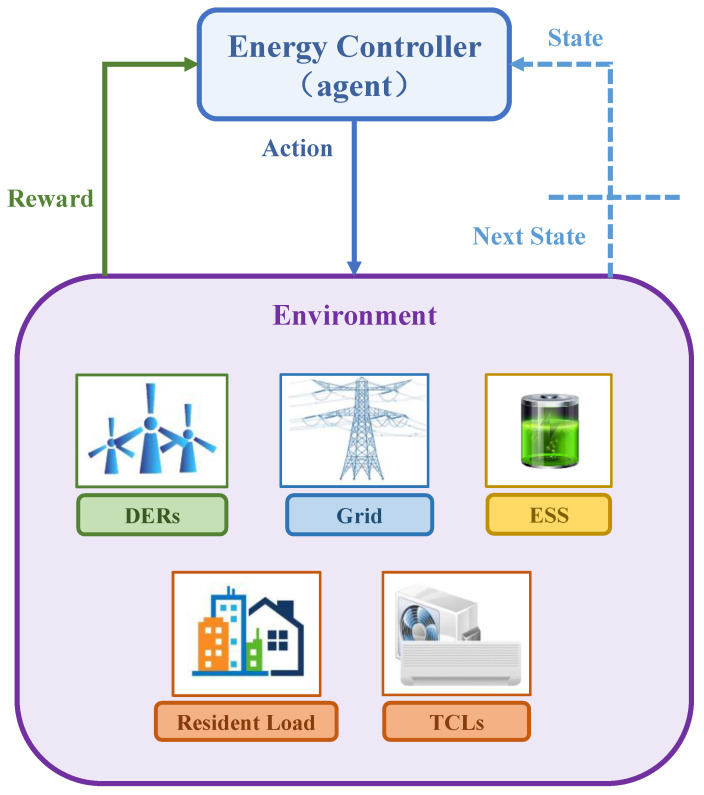
Interaction process between the energy controller and the system environment.

In this section, wind-storage cooperative model will be converted into a discrete Markov decision-making process (MDP). According to the reinforcement learning mechanism, the one-day state of the model is discretized into 24 states. In addition, the MDP in this paper takes the online environmental information as the state space, the set of command actions executed by the energy controller as the action space, and the income of electricity sellers as the reward function. The interaction process between the energy controller and the system power environment is shown in Figure 7.

### 3.1. State Space

The state space is composed of the information that the agent needs to use when making decisions at each time step t, including the controllable state component $S^{C}$, the external state component $S^{X}$, and the time-dependent component $S^{T}$. The controllable state information includes all environmental variables that the agent can directly or indirectly affect. In this study, the controllable state information is composed of TCL’s average SoC, ESS’s charge and discharge state $BSC_{t}$, and the pricing counter $C_{t}^{b}$ [33]. The external state information consists of all variables, such as the temperature information $T_{t}$, the wind power generation $G_{t}$, and the electricity price $P_{t}^{u}$. When the algorithm is implemented, the external state information directly uses the real data set, so it is assumed that the controller can accurately predict the values of three variables in the next moment. The time-dependent component information includes the information strongly related to time in the model, where $L_{b,t}$ represents the current load value based on the daily consumption mode, and t represents the hours of the day.

The state space is expressed as follows:

(19) $$s_{t}\in S=S^{C}\times S^{X}\times S^{T}$$

(20) $$s_{t}=[SoC_{t},BSC_{t},C_{t}^{b},T_{t},G_{t},P_{t}^{u},L_{b,t},t]$$

In the implementation process, the electricity price is not given directly. Firstly, the initial electricity price is set. When the price should be increased or decreased, the pricing counter $C_{t}^{b}$ will be added or subtracted by 1. Then, the electricity price becomes the initial price plus the product between $C_{t}^{b}$ and the unit electricity price.

### 3.2. Action Space

The action space consists of four parts: TCL action space $A_{tcl}$, price action space $A_{P}$, energy shortage action space $A_{D}$, and energy excess action space $A_{E}$. Among them, the TCL action space consists of four possible actions. The price action space consists of five possible actions. There are two possible actions in the energy shortage and excess action space, that is, the priority between ESS and the external power grid. Therefore, the whole action space contains 80 potential combinations of these actions, which can be expressed as follows:

(21) $$a_{t}={\left(a_{tcl},a_{P},a_{D},a_{E}\right)}_{t}$$

(22) $$a_{t}\in A=A_{tcl}\times A_{P}\times A_{D}\times A_{E}$$

### 3.3. Reward Function and Penalty Function

The main form of deep reinforcement learning (DRL) to solve problems is to maximize the reward function. The purpose of using DRL in this paper is to maximize the economic profits of the electricity sellers. Thus, the reward value can be selected as the operating gross profit, i.e., the income from selling electricity to the demand-side and the external power grid minus the cost of wind power generation and purchasing electricity from an external power grid. Therefore, the reward function $R_{t}$ and penalty function $Costs_{t}$ are defined as follows:

(23) $$R_{t}=Rev_{t}-Costs_{t}$$

(24) $$Rev_{t}=P_{t}\sum_{loads}L_{t}^{i}\;+C_{gen}\sum_{TCLs}L_{TCL}^{i}u_{b,t}^{i}+P_{t}^{d}E_{t}^{s}$$

(25) $$Costs_{t}=C_{gen}G_{t}+(P_{t}^{u}+C_{tr_{imp}})E_{t}^{P}+C_{tr_{\exp}}E_{t}^{S}$$

where $C_{gen}$ is the energy price charged to TCL, and it is also the cost of wind power generation. $G_{t}$ refers to the wind power generation amount. $P_{t}^{d}$ and $P_{t}^{u}$ are the decreased price and increased price respectively, i.e., the energy price sold to or purchased from an external power grid [25]. $E_{t}^{S}$ and $E_{t}^{P}$ are the amount of energy sold to or purchased from an external power grid, respectively. $C_{tr_{imp}}$ and $C_{tr_{\exp}}$ are the power transmission costs from the interaction with the external power grid.

## 4. Implementation Details

Before the algorithm evaluation, implementation details are given in this section.

The computer configuration and environment configuration are collected as Widows11, python3.8, tensorflow1.14; CPU is AMD R7-5800H; GPU is RTX3060; and the memory is 16 GB.

The network structure of the DQN and SARSA algorithms consists of an input layer, two fully connected hidden layers, and an output layer. The activation function of neurons is the ReLU function. In addition, in order to prevent the phenomenon of over fitting after model training, this paper applies the dropout section for neural network training. The number of neurons in the network input layer is the same as the dimension of the system state space, and the number of neurons in the output layer is the same as the dimension of the system action space. The D3QN algorithm adds a competitive network to the structure of the first two algorithms, diverting the abstract value obtained from the full connection layer into two branches. The upper path is the state value function V(s), which represents the value of the state environment itself, and the lower path is the state dependent action advantage function A(s,a), which represents the additional value brought by selecting an action in the current state. Finally, these two paths are aggregated to obtain the Q value of each action. This competitive structure can theoretically learn the value of the environmental state without the influence of action, making the practice effect better.

**Figure 8:**
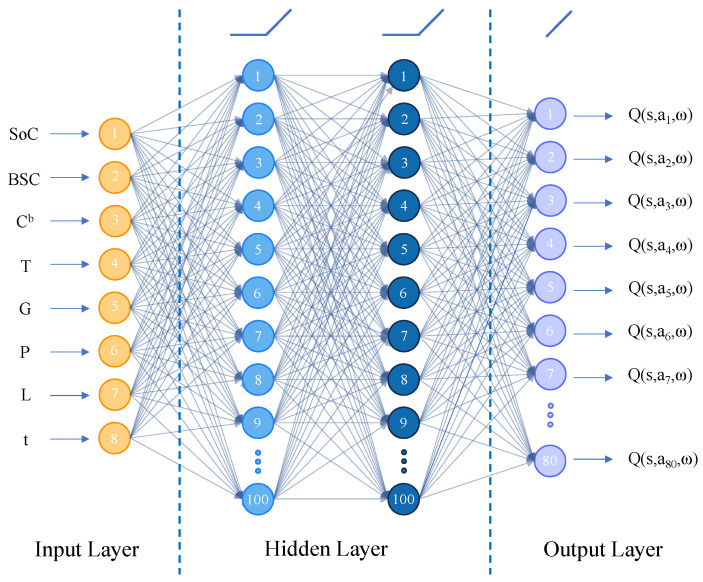
Network structure diagram of the DQN algorithm and SARSA algorithm.

**Figure 9:**
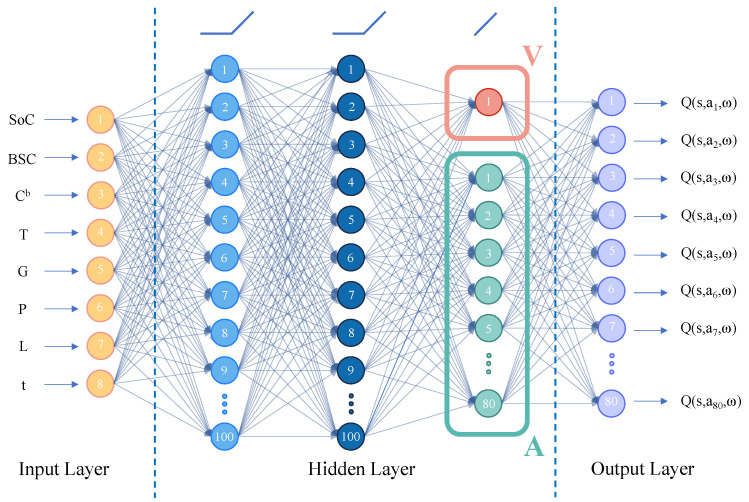
Network structure diagram of the D3QN algorithm.

In the training process of the neural network, the discarding rate in dropout is 70%, the sample storage capacity of experience playback set is 500, the scale batch used for each small batch is 200, the reward attenuation coefficient is 0.9, and the target network update interval N is 200. The detailed network structure diagrams of DQN, SARSA, and D3QN are shown in Figure 8 and Figure 9.

The proposed decision-making algorithm will be deployed in the cloud server for real-world applications. Generally, the cloud sever possesses enough computational power to execute the DL-based methods.

## 5. Algorithm Evaluation

In this section, the simulation evaluation is presented to validate the proposed control mechanism. This paper selects the wind power data of a wind farm in Finland. In the wind-storage cooperative model, the control cycle of ESS is 1 day, i.e., 24 intervals. In addition, the parameters involved in the whole system model are summarized in Table 1.

### 5.1. Comparisons of Training Results

#### 5.1.1. Penalty Value Curve

**Figure 10:**
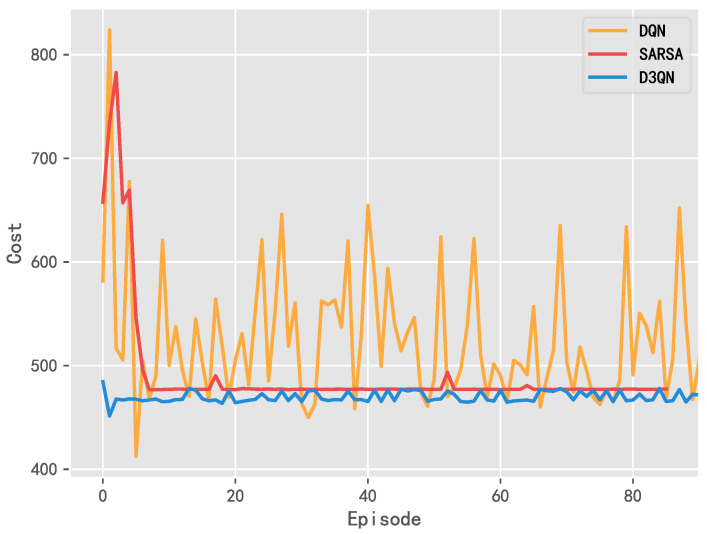
Comparison analysis of the penalty value using DQN, SARSA, and D3QN.

The penalty value is composed of the cost of wind power generation, the purchasing power from the external power grid, and power transaction. Figure 10 shows the total cost paid by the wind power producers in each training cycle (episode) during the learning process. The penalty value decreases with the increase in training times and it gradually converges.

It can be seen that the convergence performance of D3QN is superior to its rivals. Although the penalty value using DQN shows a downward and gradual convergence trend, it still vibrates obviously, which is caused by the defects of DQN. D3QN uses two Q networks to calculate the target Q value and the estimated Q value, respectively, which directly reduces the correlation and greatly improves the convergence performance.

#### 5.1.2. Reward Value Curve

**Figure 11:**
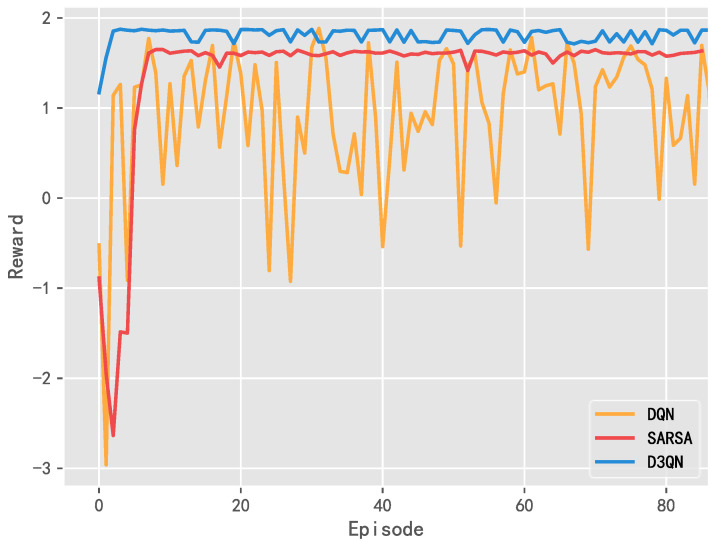
Comparison analysis of reward value curves using DQN, SARSA, and D3QN.

Figure 11 shows the reward value curve during the training process, i.e., the income obtained by the wind farm from the external environment in the operation. The specific training time, final reward mean value, and performance improvement rate between the three algorithms are summarized in Table 2. It can be seen that the final reward value of D3QN is higher than that of the other two algorithms, so the overall performance of the system model based on D3QN has been improved.

### 5.2. Comparison of Application Results

#### 5.2.1. 10 Day Revenue Comparison

**Figure 12:**
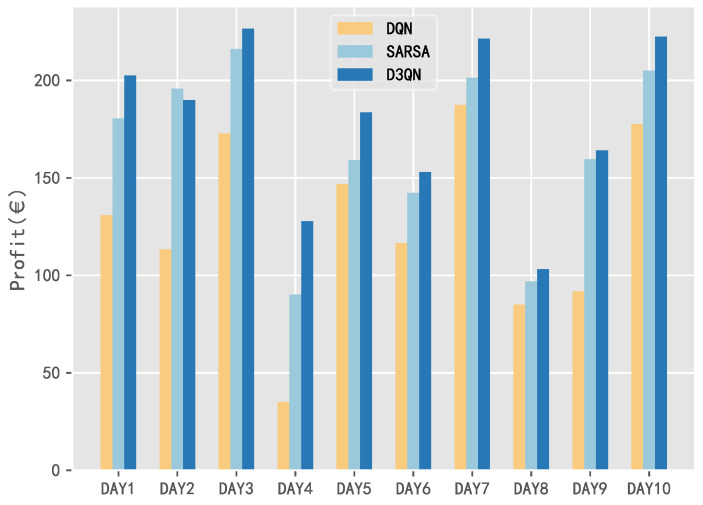
Comparison analysis of the daily income with three DRL algorithms for 10 days in a year.

In order to give a more intuitive understanding of the performance difference for DQN, SARSA, and D3QN, this section selects the data from 10 days in a year, and analyzes the daily total profit obtained by the system model with the three algorithms, as shown in Figure 12.

It can be seen that the daily income using SARSA and D3QN is higher than that of DQN within 10 days. Moreover, the total profit of D3QN is better than that of SARSA in 9 out of 10 days, which also validates the superiority of D3QN.

#### 5.2.2. Daily Electricity Trading Comparison

**Figure 13:**
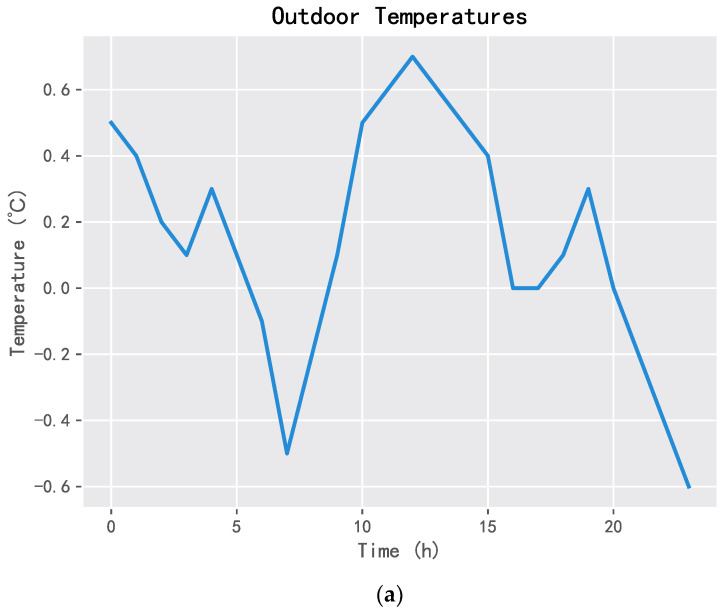

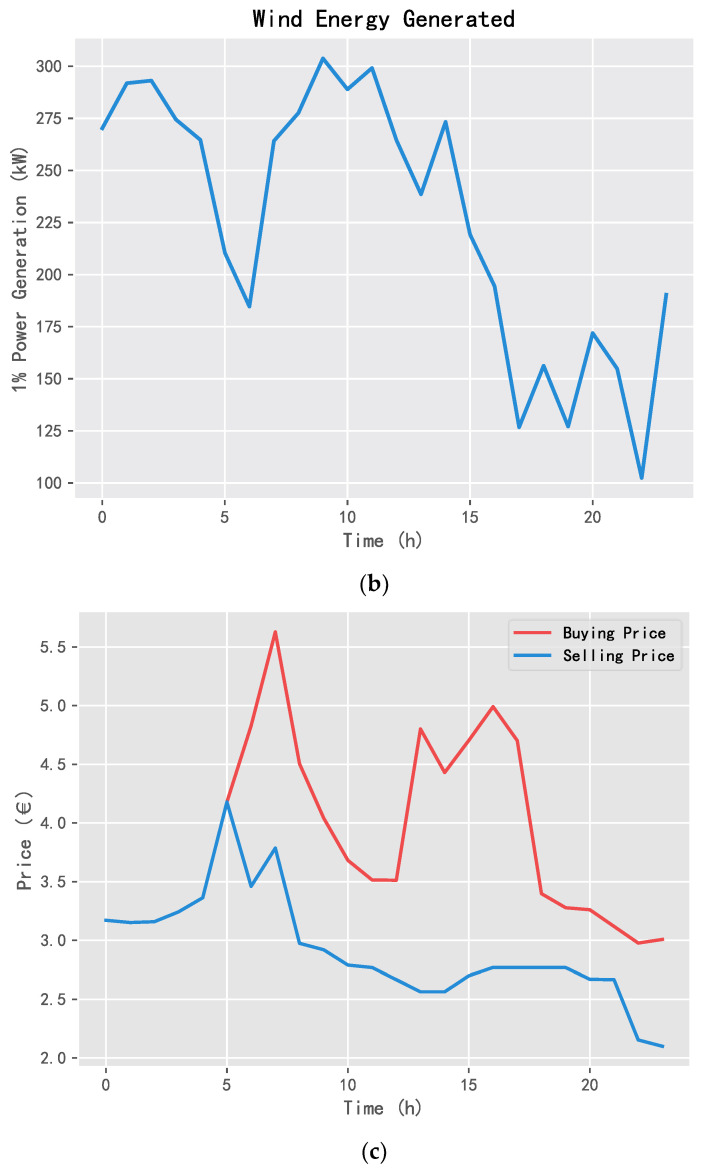

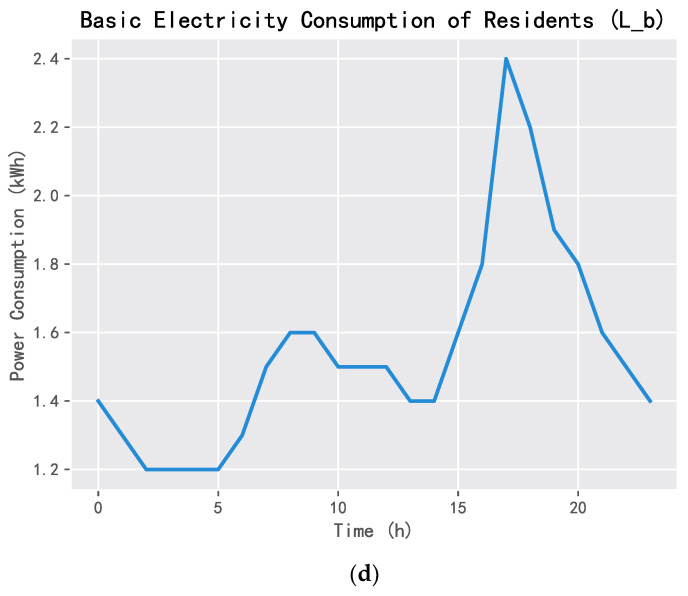
Environmental data of one-day: (**a**) outdoor temperature, (**b**) energy generated, (**c**) electricity prices, and (**d**) residential loads.

This section will compare the behavior of the three algorithms in the specific one-day. The one-day data of the environment is shown in Figure 13, including the outdoor temperature, wind power generation, electricity prices, and residential load.

**Figure 14:**
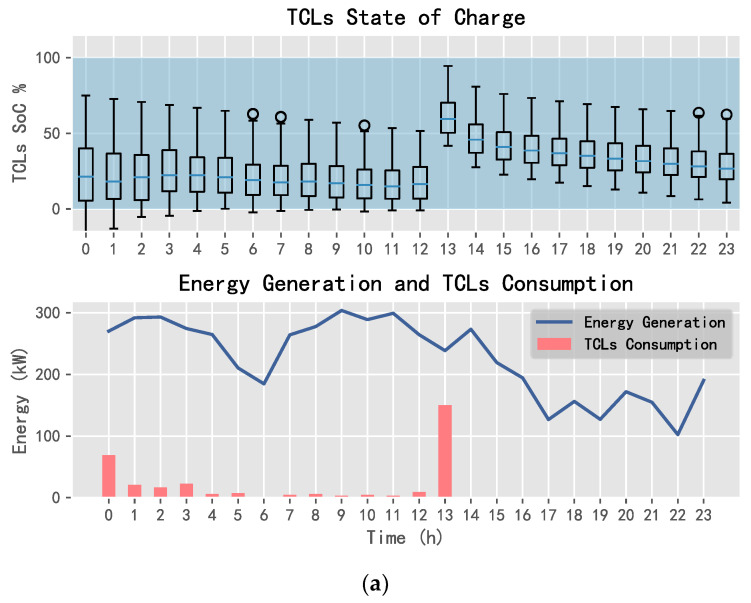

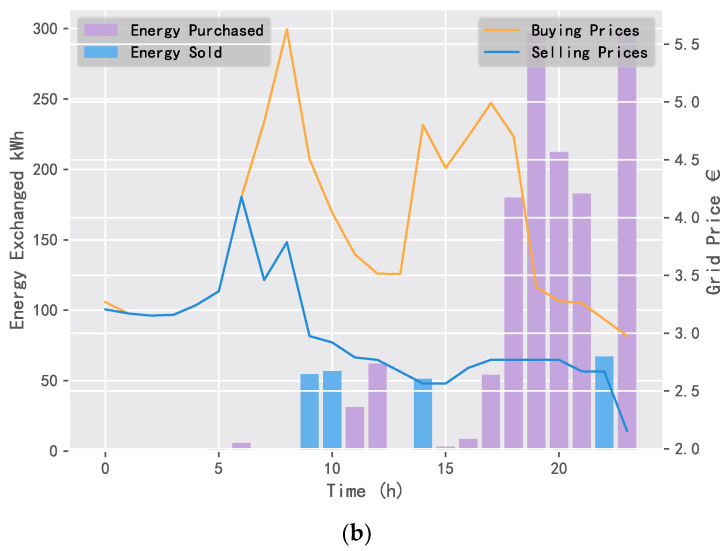
TCLs status and power exchange using DQN: (**a**) TCLs and (**b**) power exchange.

**Figure 15:**
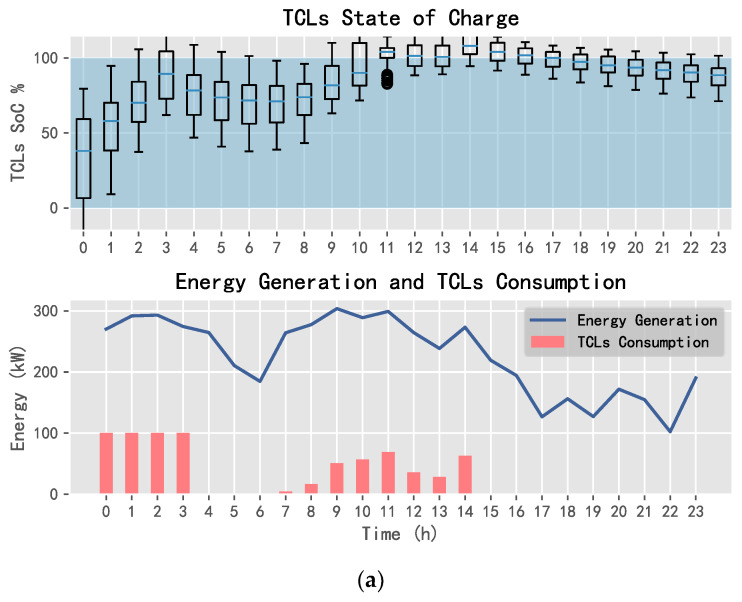

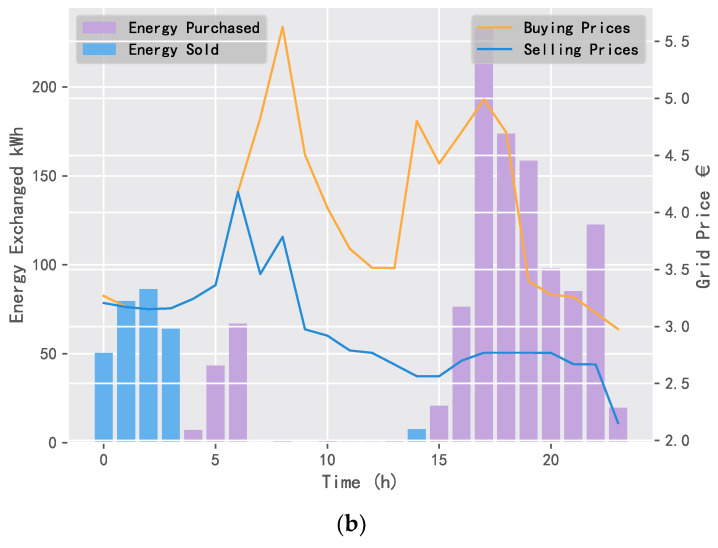
TCLs status and power exchange using SARSA: (**a**) TCLs and (**b**) power exchange.

**Figure 16:**
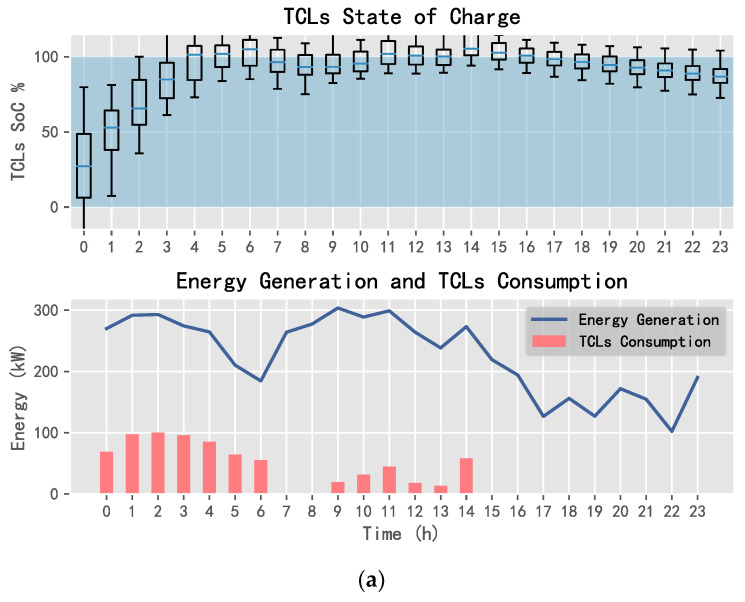

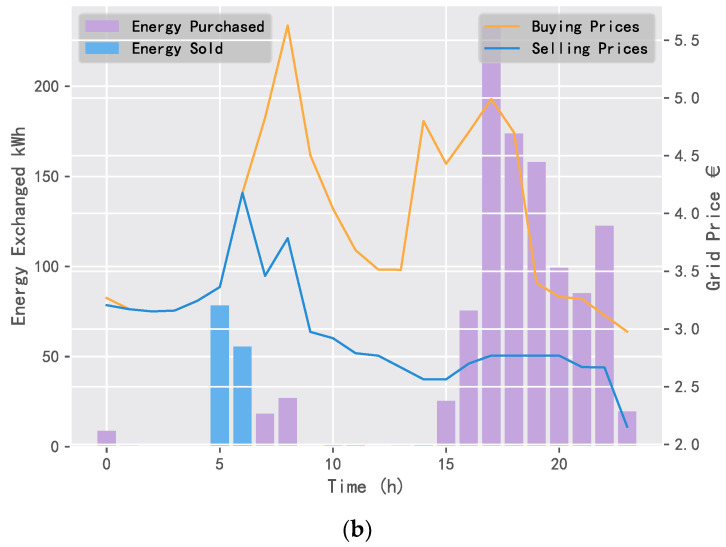
TCLs status and power exchange using D3QN: (**a**) TCLs and (**b**) power exchange.

Using DQN, SARSA, and D3QN, one can obtain the energy allocation results of TCLs, the purchased energy, and the sold energy, as shown in Figure 14, Figure 15 and Figure 16.

In Figure 14, Figure 15 and Figure 16, the SoC of TCLs reflects the change in indoor temperature for residents. This paper sets the constant temperature range of TCLs as 19~25 °C. When the charging state of TCLs is 0%, it means that the indoor temperature of residents is less than or equal to 19 °C; when the charging state is 100%, it means that the indoor temperature is greater than or equal to 25 °C. It can be seen that SARSA and D3QN can allocate sufficient energy to TCLs when the wind power generation is sufficient, where its state can reach saturation as soon as possible, such that the system can keep the room temperature stable, and gives residents a warm and comfortable experience. In addition, SARSA selects multiple transactions to ensure the income, and D3QN decisively sells a large amount of power to obtain more income when wind energy is sufficient and the electricity price is the highest.

#### 5.2.3. Computational Efficiency Comparison

In order to demonstrate the computational efficiency of the proposed D3QN, the training time, decision-making time, the number of trainable parameters, and performance improvement rate are summarized in Table 3. It takes 196.0111 and 415.5845 s for DQN and SARSA to reach convergence, respectively, while the proposed D3QN takes 244.1469 s. Furthermore, although D3QN possesses the largest number of trainable parameters, the decision-making time of D3QN is close to the other two algorithms, which demonstrates that D3QN can be implemented in real-world applications. From Table 3, one can conclude that the computational cost of D3QN is slightly larger than DQN and SARSA, which is still in an acceptable range. However, it should be noted that it is mainly because of many trainable parameters. Moreover, the performance improvement rate of D3QN is the biggest, which is an important criterion to evaluate different algorithms. Generally, it is worth increasing some computational complexity while the performance can gain enough improvement.

## 6. Conclusions

Considering external conditions such as wind energy resources, demand response load, and market electricity price, this paper puts forward a new research method of wind-storage cooperative decision-making based on the DRL algorithm. The main work of this paper is summarized as follows:

(1) This paper proposes a new wind-storage cooperative model. Based on the conventional model including wind farms, energy storage systems, and external power grids, this paper also takes into account a variety of flexible loads based on demand response, including residential price response loads and thermostatically controllable loads (TCLs). Meanwhile, this model also can be applied to other renewable energy sources, such as photovoltaic power generation, hydroelectric power generation, and thermal power generation.

(2) This paper proposes a new wind-storage cooperative decision-making mechanism using D3QN, which takes the energy controller as the central allocation controller of the system energy, realizing the direct control of TCLs and the indirect control of the residential price response load, and the management of priority between ESS and the external power grid in the case of sufficient or insufficient energy.

(3) It is worth mentioning that the application of the D3QN algorithm is a new attempt in the research field of wind-storage cooperative decision-making. Based on the historical data of wind farm and market electricity prices, the effectiveness of D3QN in dealing with the wind-storage cooperative decision-making problem is verified, and the superior performance of D3QN is also analyzed.

## Figures and Tables

**Table 1:** Parameters in the system model.

Parameter	Value
ESS	
$\eta_{c}$	0.9
$\eta_{d}$	0.9
$C_{\max}$	250 kW
$D_{\max}$	250 kW
$B_{\max}$	500 kWh
DER	
$G_{t}$	1% of the hourly wind power generation (kW)
$C_{gen}$	32 €/MW
Power grid	
$P_{t}^{d}$	Reduced electricity prices
$P_{t}^{u}$	Increased electricity prices
$C_{tr_{imp}}$	9.7 €/MW
$C_{tr_{\exp}}$	0.9 €/MW
TCL	
$N_{tcls}$	100 (Number of TCL)
$T_{t}^{0}$	Outdoor temperature hourly
$C_{a}^{i}$	N(0.004,0.0008)
$C_{m}^{i}$	N(0.3,0.004)
$q^{i}$	N(0,0.01)
$L_{TCL}^{i}$	N(1.5,0.01) (kW)
$T_{\min}^{i}$	19
$T_{\max}^{i}$	35
Load	
$N_{L}$	150
$N_{L}$	Basic load of residents
$\lambda_{i}$	N(10,6) (kW)
$\beta_{i}$	N(0.4,0.3)
Other parameters	
D	24
$\delta_{t}$	{−2,−1,0,1,2}
cst	1.5
threshold	4
$P_{market}$	5.48 €/kW
Parameters involved in the algorithm	
$N_{A}$	80
$A_{tcl}$	{0,50,100,150}
$A_{P}$	{−2,−1,0,1,2}
$A_{D}$	{ESS,Grid}
$A_{E}$	{ESS,Grid}
γ	0.9
t	1 h

**Table 2:** Training results between three algorithms.

Algorithm	Training Time (s)	Average Value of Final Reward	Performance Improvement Rate
DQN	196.0111	1.2443	-
SARSA	415.5845	1.6239	30.5%
D3QN	244.1469	1.7909	43.93%

**Table 3:** Computational efficiency comparison between three algorithms.

Algorithm	Training Time (s)	Decision-Making Time (s)	The Number of Trainable Parameters	Performance Improvement Rate
DQN	196.0111	0.347	8980	-
SARSA	415.5845	0.354	19,080	30.5%
D3QN	244.1469	0.390	27,160	43.93%

## Data Availability

Data sharing are not applicable.
